# Pharmacology, Toxicology, and Rational Application of Cinnabar, Realgar, and Their Formulations

**DOI:** 10.1155/2022/6369150

**Published:** 2022-09-27

**Authors:** Huifang Guan, Yan Xu, Chunyu Ma, Dexi Zhao

**Affiliations:** ^1^College of Traditional Chinese Medicine, Changchun University of Chinese Medicine, Changchun 130117, China; ^2^College of Pediatrics, Henan University of Chinese Medicine, Zhengzhou, Henan 450000, China; ^3^Department of Encephalopathy, The Affiliated Hospital of Changchun University of Chinese Medicine, No. 1478 Gong-Nong Road, Changchun, Jilin 130021, China

## Abstract

**Materials and Methods:**

A search was performed for the literature on cinnabar and realgar in PubMed, the Chinese Pharmacopeia, Google, and other sources. The search included studies using single herbs, traditional formulations, or novel dosage forms.

**Results:**

Cinnabar and cinnabar formulas exhibit good efficacy for sedation, sleep improvement, anxiety alleviation, and brain protection. However, previous studies on neurotransmitters have reached different conclusions, and detailed pharmacological mechanisms are lacking. Realgar and its formulas exert promising antitumor activity through regulation of cell cycle arrest, intrinsic and extrinsic apoptosis, induction of differentiation, autophagy, metabolic reprogramming, matrix metalloproteinase-9 (MMP-9) signaling, and reactive oxygen species (ROS) generation. In addition, realgar can be used to treat a variety of refractory diseases by regulating immunity and exerting antibacterial, antiviral, and other effects. However, the existing pharmacological research on the use of realgar for epidemic prevention is insufficient, and animal experiments and research at the cellular level are lacking. Inappropriate applications of cinnabar and realgar can cause toxicity, including neurotoxicity, liver toxicity, kidney toxicity, and genotoxicity. The toxicological mechanism is complex, and molecular-level research is limited. For clinical applications, theory and clinical experience must be combined to guide scientific and rational drug use and to achieve reduced toxicity and increased efficacy through the use of modern preparation methods or combined drugs. Notably, when cinnabar and realgar are used to treat targeted diseases, these agents have a bidirectional effect of “treatment” and “toxicity” on the central nervous system in pathological and normal states. The pharmacological and toxicological mechanisms need to be elucidated in greater detail in the future. Overall, systematic research is needed to provide a basis for better promotion of the rational use of cinnabar and realgar in the clinic.

**Conclusion:**

Mineral medicines are multicomponent, multiactivity, and multitargeted substances. The pharmacology and mechanisms of the toxicity and action of realgar and cinnabar are extremely complex. A number of Chinese medicinal preparations of realgar and cinnabar have demonstrated unique efficacy in the treatment of refractory diseases.

## 1. Introduction

Mineral medicines include raw minerals (cinnabar, calamine, natural copper, realgar, gypsum, etc.), processed products of raw mineral materials (calomelas, mirabilite, etc.), and animal bones or fossils of animal bones (bone or teeth fossils of large mammals) and are characteristic of traditional medicines used in China.

Throughout history, mineral medicines have played an important role in traditional Chinese medicine (TCM). TCM has a long application history with the accumulation of rich clinical experiences and is still widely used today. The earliest record of the roles of mineral medicines is documented in The Classic of Mountains and Seas, which states that taking mineral medicines can “make people healthy and live longer.” In addition, the modern 2020 edition of the Pharmacopoeia of the People's Republic of China (2020ChP) [1] affirms the wide range of clinical applications of mineral medicines in internal medicine, orthopedics, gynecology, and otorhinolaryngology. Cinnabar and realgar are two mineral medicines commonly used in the clinic with confirmed curative effects. However, their safety is of great public concern. Therefore, these medicines will be the focus of this minireview.

Cinnabar is described by the 2020ChP [1] as follows: “This product is the sulfide mineral cinnabar of the cinnabar family, which mainly contains Mercury sulfide (≥96%), which has the effects of clearing away the heart-fire and sedative, relieving uneasiness of body and mind, improving eyesight, and detoxifying.” Cinnabar is a critical sedative component of numerous Chinese prescriptions [2], such as Zhusha Anshen Pill (ASAS) and Baizi Yangxin Pill (BZYX). In addition, modern research has found that cinnabar has antianxiety, tranquillizing, antioxidative stress, and anti-brain-damage effects, and the mineral is used in compound preparations for the treatment of insomnia, anxiety disorders, brain trauma, stroke, and neuroinflammation [3].

Realgar is described by the 2020ChP [1] as a sulfide mineral of the realgar family that mainly contains arsenic disulfide (As₂S₂) and has the functions of “detoxifying and killing insects, drying dampness and removing phlegm, and treating malaria.” Modern research has further revealed that realgar can be combined with other drugs to treat a variety of blood system diseases [4], including acute promyelocytic leukemia (APL) [5], myelodysplastic syndrome (MDS) [6–9], and lymphoma. Realgar has also shown significant pharmacological effects against primary or metastatic cancers, such as breast cancer (cells) [4, 10, 11], cervical cancer [12], lung cancer [13], osteosarcoma [14], gastric cancer [15], oral cancer (cells) [16], and liver cancer [17], in nonclinical studies. In addition, realgar exhibits good immunoregulatory, antiviral, and antiepidemic effects, which may provide new ideas for the treatment of viral infectious diseases.

In recent years, the occurrence of phytotoxicity incidents caused by the improper use of mineral drugs has increased the concern regarding their toxicity. Cinnabar and realgar have become popular discussion topics because their main components (Mercury and arsenic) are known toxic metals/metalloids. Some have even questioned the necessity of heavy metal minerals, such as cinnabar and realgar, in medicinal formulas.

Several studies have found that different forms of Mercury can be absorbed through the gastrointestinal tract, respiratory tract, and skin and that excess levels of Mercury and its compounds have acute or chronic toxic effects on the human body [18–23]. Arsenic toxicity is associated with liver tumors, diabetes, and cardiovascular, neurological, and other diseases [24–26]. However, some in vivo experiments in mice have indicated that cinnabar does not significantly affect transporter genes in the liver and kidneys [27, 28]. This contradiction underscores the need for further research on the pharmacology and toxicology of cinnabar and realgar.

When discussing Mercury toxicity and arsenic toxicity, it is necessary to distinguish the chemical forms. In traditional Chinese medicine, Mercury and arsenic are used orally in sulfide forms [29]. The chemical forms of cinnabar and realgar are significant determinants of their activity and toxicity. Both cinnabar and realgar are far less toxic than the well-known Mercury [21, 30–32] and arsenic [33]. In addition, studies have demonstrated that cinnabar (*α*-HgS) differs from environmental Mercury (HgCl_2_, MeHg) based on its bioavailability, intestinal transport levels, distribution, metabolism, elimination, and toxicity [29, 32]. Similarly, realgar (As₂S₂, As_4_S_4_) differs from sodium arsenate (As^5+^) and sodium arsenite (As^3+^) in structure, disposition, efficacy, and toxicity [29].

Thousands of years of clinical practice have suggested the effectiveness of cinnabar and realgar. However, the exact pharmacodynamic and toxic mechanisms of these minerals and how to optimize their clinical applications remain unclear; an in-depth research with modern research techniques is needed. A literature search of the use of Chinese materia medica in the treatment of diseases reveals that cinnabar and realgar are seldom used alone. These agents are commonly used in multiherb/metal mixtures. Therefore, we recommend that analyzing the mechanisms of clinically effective formulations at the molecular, cellular, and organismal levels represents a potentially effective method for assessing the value of traditional medicines.

In this article, we briefly summarize the main pharmacological and toxicological characteristics and usage specifications of the mineral medicines cinnabar, realgar, and their commonly used preparations, aiming to provide a theoretical basis for the rational and safe clinical use of cinnabar and realgar.

## 2. Cinnabar

For thousands of years, cinnabar has been used to treat different diseases [2]. According to the 2020ChP [1], approximately 10%–30% of Chinese compound prescriptions contain cinnabar. Representative prescriptions include ASAS and Baizi Yangxin Pill. According to traditional Chinese medicine, cinnabar taken orally can clear away heart fire, exert sedative effects, relieve uneasiness in the body and mind, improve eyesight, and detoxify. Modern research has shown that cinnabar can improve sleep, combat anxiety and oxidative stress, and protect the brain from damage.

However, the use of cinnabar in clinical practice has been controversial because it contains the heavy metal Mercury. Epidemiological investigations and animal experiments have shown that excessive intake of cinnabar can exert toxic effects on the kidneys, liver, and nervous system. Therefore, the pharmacology and toxicology of cinnabar and traditional medicines containing cinnabar have attracted attention.

### 2.1. Pharmacological Research on Cinnabar Alone

#### 2.1.1. Sedative and Anxiolytic Mechanisms

Cinnabar has long been used in combination with other Chinese materia medica as a sedative for more than 2000 years [2]. The sedative effect of cinnabar has been verified in animal experiments. For example, studies have shown that, in mice administered low-dose cinnabar (10 mg/kg/d) for 11 weeks, motor activity is decreased, and pentobarbital-induced sleeping time is prolonged, suggesting that cinnabar has sedative effects [34]. Wang et al. confirmed the anxiolytic effect of cinnabar on anxiety-like behaviors in mice using the elevated plus maze test; this neuropharmacological effect may have been similar to the sedative and soothing effects of cinnabar. The results suggest that cinnabar exerts anxiolytic effects when administered chronically at effective doses and is associated with reduced brain serotonin (5-HT) levels [35].

#### 2.1.2. Antioxidative Stress and Brain Injury Protection Mechanisms

Oxidative stress plays a key role in neuronal death and underlies neurodegenerative diseases. Thus, antioxidants can play important roles in treating several neurodegenerative diseases [36]. Reactive oxygen species (ROS) are markers of oxidative stress that can indicate the state of redox balance in the body.

One study confirmed that cinnabar can reduce the disappearance of antioxidant enzymes and the overproduction of ROS through regulation of the 5-HT metabolism pathway under hypoxic conditions at the cellular level and in zebrafish in vivo, ultimately alleviating hypoxia-induced oxidative stress and significantly improving the survival of neuronal cells under high-ROS conditions [37]. This finding highlights one of the potential mechanisms by which cinnabar exerts neuroprotective effects.

**Figure 1:**
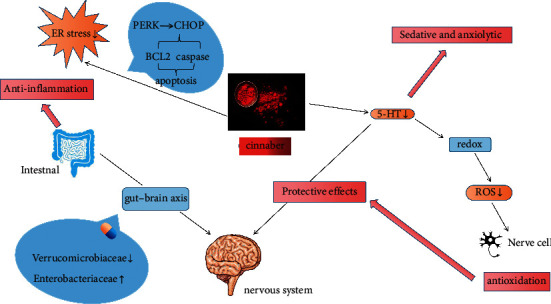
Pharmacological mechanism of cinnabar.

In the endoplasmic reticulum (ER) stress response, protein kinase RNA-like ER kinase (PERK) induces the activation of C/EBP homologous protein (CHOP). Activated CHOP then induces apoptosis via the apoptosis receptor BCL2 family proteins and the death receptor caspase family proteins of the downstream mitochondria-dependent pathway [38]. Low cinnabar concentrations induce antioxidative and antiapoptotic effects on cells and inhibit intracellular stress responses. The possible mechanism involves blockade of ER stress-induced apoptosis via downregulation of PERK expression and indirect downregulation of CHOP activation [39]. In an emerging microbiome study, cinnabar reduced neuronal stress and inflammation through the gut–brain axis by altering the microbiome structure via reductions in Verrucomicrobiaceae and increases in Enterobacteriaceae, suggesting that HgS-containing traditional medicines can mechanistically target gut microbiota to exert their therapeutic effects [40]. The pharmacological mechanism of cinnabar is presented in Figure 1.

#### 2.1.3. Research on Cinnabar Formulas

Given its good curative effect, cinnabar has been widely used as a sedative ingredient in TCM prescriptions. However, the mechanisms of cinnabar and its various formulations have been the subject of few studies. Some pharmacological studies on the use of cinnabar and its formulations for the treatment of insomnia have been reported in the Chinese literature but are not available in PubMed. One study found that the formula ASAS, which includes cinnabar as the main therapeutic ingredient, exhibits good sedative and antianxiety effects. A study on ASAS in rats with conditioned fear showed that this pill can antagonize conditioned fear; the antagonistic effect is mainly reflected in the fading stage of fear memory and may be involved in regulating the content of monoamine neurotransmitters and the expression of c-Fos protein in the basolateral amygdala. This mechanism increases the efficiency of fear extinction and improves sleep in rats [41]. The mechanism is related to hippocampal neuron protection, hippocampal synaptic structure regulation, and functional plasticity [42]. In the sleep phase, moderate and high doses of a decoction of ASAS obviously decrease the duration of wakefulness and increase the total sleep time. A moderate dose of the decoction obviously increases the duration of slow-wavestage-1 sleep, whereas high doses of the decoction obviously increase the duration of slow-wavestage-2 sleep. Although low doses do not decrease the duration of wake, they can increase the duration of slow-wavestage-2 sleep [43]. The mechanism may be related to inhibition of the production of 5-HT and norepinephrine (NE) monoamine neurotransmitters in the ventrolateral preoptic area [44] that increase the content of *γ*-aminobutyric acid in the brain [45].

### 2.2. Toxicological Research on Cinnabar

Cinnabar is a natural medicinal substance containing metallic Mercury [46]. Regarding toxicology, cinnabar has known toxic effects on the kidneys, liver, and nervous system. Wei et al. [47] applied a nuclear magnetic resonance metabolomics method and found that cinnabar altered biochemical indexes with time-anddose-dependent effects. Proteomics has shown that therapeutic and toxic doses of cinnabar affect different pathways and potential targets in the mouse cerebral cortex [48]. In addition, a study found that the cerebellum is more sensitive to cinnabar treatment than the cerebrum [19]. Some studies have revealed that exposure to a high dose (1.0 g/kg/day for 7 or 14 consecutive days) of cinnabar or HgS causes ototoxicity [20] and neurotoxicity [21, 49], whereas 0.01 g/(kg day) causes no toxic effects [21, 49].

Cinnabar toxicity may occur because high dose or long-term use of Chinese compound preparations containing cinnabar leads to the accumulation of Mercury in the kidneys, resulting in kidney damage, such as kidney inflammation and mild fibrosis [22]. Cinnabar may also cause proximal tubular damage, which may be related to the activation of the renal tubular apoptosis pathway and the expression of organic anion transporters (OATs) and the expression of tubular basal transporters organic anion transporter OAT1 and OAT3 [23, 50]. One study found that the gut microbiota is a potential target for the dual effects of cinnabar and that oligopeptide transporter 1 may represent an important transporter of cinnabar into the intestinal epithelium [32]. Under the influence of human intestinal bacteria, cinnabar is transformed into nontoxic products (mercuric polysulfides) rather than methylmercury [51].

Although long-term use of cinnabar can cause a series of toxic side effects, scholars have found that cinnabar is indeed much less toxic than other mercury-containing compounds, such as HgCl_2_ [30, 32] and MeHg [21, 31].

This reduced toxicity may be related to the downregulation of oligopeptide transporter 1 mRNA and protein expression by cinnabar [32], which affects the intestinal absorption and transport of cinnabar; its dissolution, absorption, distribution, and accumulation in vivo; and the molecular configuration of Mercury compounds, particle size, and biological activity of the gastrointestinal tract [52–54].

The toxic effects of cinnabar have varied greatly in different experiments. In addition to disparities in the administered dose, different methods of cinnabar processing may also have contributed to the different toxicities. Ancient traditional Chinese medicine practitioners stressed that the washing (called Shui-Fei) method during cinnabar processing was extremely sensible, as it avoided high temperatures and reduced the levels of soluble toxic elements. However, different researchers have used different medicinal materials in their toxicological studies on cinnabar, and only a few studies have emphasized the use of washing methods, which may be another important reason explaining the inconsistently experimental results. Given that the detoxification methods of cinnabar and realgar are similar, the final statement is unified.

## 3. Realgar

Realgar contains >90% arsenic sulfide (As₂S₂) and is widely used externally and internally in many traditional medicine recipes in China. The 2020ChP [1] contains more than 30 types of formulas containing realgar with representative formulas including the Niuhuang Jiedu Pill, Niuhuang Qingxin Pill, and Angong Niuhuang Pill. The functions of realgar are mostly “detoxifying and killing insects, drying dampness and removing phlegm, and treating malaria” [1]. Realgar is used for various diseases, such as “carbuncles, swelling, sores, snake bites, ascariasis, epilepsy, and malaria” [1].

Recent studies have found that realgar exhibits significant anticancer activity. The possible antitumor mechanism may be closely related to the regulation of cell cycle arrest, intrinsic and extrinsic apoptosis, induction of differentiation, autophagy, metabolic reprogramming, matrix metalloproteinase-9 (MMP-9) signaling, and ROS generation [4, 10].

However, 90% of realgar is As₂S₂, which exhibits toxic effects characteristic of heavy metals; thus, the clinical application of realgar is controversial. Epidemiological investigations and animal experiments have shown that long-term arsenic exposure can lead to language, cognitive, behavioral, and hearing and motor impairments, as well as mental retardation and neurological deficits in severe cases. Even when arsenic exposure ceases, central nervous system function does not quickly return to normal [55]. Therefore, the pharmacology and toxicology of realgar and traditional medicines containing realgar have attracted extensive attention.

### 3.1. Pharmacological Research on Realgar

#### 3.1.1. Pharmacological Research on Realgar Alone

*(1) Antitumor Mechanism*. Induction of tumor cell apoptosis is the main mechanism of action of realgar in the treatment of tumors, and these effects can be achieved through a variety of ways. Studies have demonstrated a link between oxidative stress and tumor cell apoptosis [56]. To improve the activity of realgar, some researchers have used intrinsic biotransformation pathways in microorganisms to obtain a realgar transformation solution (RTS). RTS activates p53-mediated cell cycle arrest and apoptotic pathways by inhibiting the cellular antioxidant defense system and massive accumulation of ROS in tumor cells and by inducing apoptosis and interrupting G2/M progression in HepG2 cells to inhibit the growth of tumor cells and promote apoptosis [17]. Similar mechanistic studies have shown that the expression of proapoptotic proteins, including caspase-7 and caspase-8, is activated under the action of As₂S₂ to trigger tumor cell apoptosis [10]. The results of another study suggest that As₂S₂ inhibits cell viability and induces apoptosis and cell cycle arrest in both MCF-7 and MDA-MB-231 cell lines by regulating the expression of key proteins involved in related pathways [4].

Cell cycle-regulated gene mutations play important roles in tumorigenesis. Cell cycle arrest provides cells with additional time to repair damage, thereby reducing the occurrence of mutations and tumor development. In a molecular study on the use of As₂S₂ in the treatment of APL, As₂S₂ was found to induce the accumulation of NB4-R1 cells in S and G(2)/M phases [57]. This effect may be related to the regulation of the expression of related proteins, including cyclin B1 and cell division cycle protein 2 [10].

Induction of differentiation is a common strategy in tumor therapy. Realgar induces multilineage differentiation in the human promyelocytic leukemia cell line HL-60, thereby exerting anticancer effects. Myeloid differentiation of HL-60 cells is induced via the p38 mitogen-activated protein kinase-related signaling pathway [58], and monocytic differentiation of HL-60 cells is induced via serine/threonine protein phosphatase-related pathways [59]. Realgar simultaneously induces apoptosis and differentiation in both the all-trans retinoic acid- (ATRA-) sensitive NB4 and ATRA-resistant MR2 cell lines [60]. The following specific mechanisms may be involved: (1) binding of realgar to the pML/RAR*α* fusion protein, making it ineffective; (2) enhancement of the level of cellular ubiquitin by realgar; (3) promotion of pML/RAR*α* fusion protein ubiquitination; (4) formation of a covalent enzyme system; and (5) entry into the protease system for degradation into short peptides and loss of activity [5].

Autophagy is an important cell biological process induced by multiple forms of cellular stress, including nutrient or growth factor deprivation, hypoxia, and ROS [61]. Excessive levels of autophagy damage body tissues and cells and disrupt homeostasis [62]. Realgar can induce autophagy in endometrial cancer JEC cells as one of its anticancer mechanisms [63]. In addition, realgar preparations can induce autophagy via upregulation of LC3 and p62/SQSTM1 [64] and inhibit the Akt/mTOR signaling pathway [14].

Under hypoxic conditions, hypoxia-inducible factor (HIF-1) promotes the metabolic reprogramming of tumor cells and induces acidification of the tumor extracellular environment, which helps tumor cells obtain energy and survive [65]. However, nanorealgar inhibits tumor growth in vitro and in vivo by repressing metabolic reprogramming. This inhibitory effect is potentially related to the downregulation of HIF-1*α* expression via the PI3K/Akt/mTOR pathway [13].

MMP-9 is one of the most complex matrix metalloproteinases. In tumor cells, MMP-9 contributes to the remodeling of extracellular matrix proteins, providing a microenvironment for tumorigenesis. MMP-9 degrades basal type IV collagen near tumor cells and then invades other normal tissues, thereby inducing cancer invasion and metastasis [66]. However, As₂S₂ treatment inhibits MMP-9 protein expression [10].

Studies have shown that As₂S₂ inhibits the proliferation of colon cancer cells by regulating the nuclear factor of activated T cells (NFAT) pathway [15, 67]. Its inhibitory effect is related to a signaling pathway related to vascular endothelial growth factor 2 [68].

These results suggest that realgar preparations have broad-spectrum scavenging effects in tumor cells. In cells and tissues, regulation is a complex process involving multiple signaling pathways, and the pathways in which realgar is involved require further study.

*(2) Immunoregulatory and Antibacterial Mechanisms*. Severe systemic lupus erythematosus (SSLE) refers to a manifestation of systemic lupus erythematosus (SLE) involving important organs. Patients with SSLE often experience multiple complications simultaneously, treatment difficulties, and worse prognoses [69]. Studies have shown that realgar can be used to treat severe forms of SLE, such as lupus nephritis, and that the mechanism may involve downregulation of the expression of phosphorylated signal transducer and activator of transcription 1 [70].

Realgar promotes apoptosis of B cells from MRL/lpr mice by increasing Ca^2+^ concentrations, reducing surface molecule activation, stimulating surface factor expression, and restraining its activation to reduce the production of antibodies, reduce MRL/lpr mouse autoimmune injury, and slow down the SLE course [71]. In addition, in *Caenorhabditis elegans*, realgar can alleviate the infection of wild-type N2, *glp*-4 mutant, and *daf*-2 mutant nematode strains by inducing both immune and protective responses and significantly increasing antibacterial effector levels, which leads to pathogen elimination [72, 73].

*(3) Prevention and Treatment of Epidemics*. Epidemic diseases are highly contagious and dangerous. Historically, realgar has played an important role in the prevention and treatment of epidemics in China in past dynasties. However, pharmacological research on realgar use for epidemic prevention and treatment is scarce. According to a recent literature search, realgar was the drug most frequently used by physicians for epidemic prevention in past dynasties [74]. Wearing colorful sachets containing realgar, drinking realgar wine, and sprinkling realgar on walls and doors during the Dragon Boat Festival represent traditional public uses. Realgar works primarily by killing pathogens (disinfecting) and expelling pathogens (detoxifying) [75].

External use through various methods (i.e., burning, externally coating, wearing, stuffing, sneezing, and tears) can block the transmission of the epidemic pathogen. Realgar can also be taken internally in the form of pills, medicinal liquors, etc. and can be combined with cinnabar for epidemic prevention. Ruan [76] and others believed that coronavirus disease 2019 (COVID-19) was caused by a “cold-damp pestilential pathogen” invading the human body according to climatic, seasonal, and regional characteristics. Based on syndrome differentiation, realgar and other pungent and warm (Xin Wen) products were chosen to relieve the cold-damp poison. Similarly, Changchun University of Traditional Chinese Medicine also provided guidance that the new strains of coronavirus, mainly Omicron, that broke out in China in 2022 were caused by a cold-damp pestilential pathogen and suggested that pungent and warm (Xin Wen) products should be used. Compared with the epidemic prevention and treatment methods of modern medicine, those of traditional Chinese medicine have broad-spectrum immune effects and strong universality, are forward-looking, and achieve the purpose of disease prevention. Research on the prevention and treatment of diseases with realgar may provide new ideas for the use of TCM for the prevention and treatment of modern epidemics. However, the antiepidemic mechanism of action of realgar requires further study.

**Figure 2:**
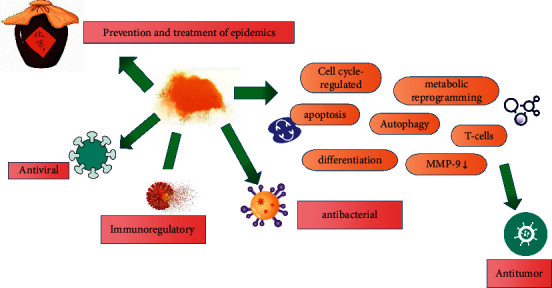
Pharmacological mechanism of realgar.

*(4) Antiviral Mechanism*. In an experiment using acyclovir as a positive control, nanorealgar showed good anti-herpes simplex virus type II activity when administered in 3 modes (pretreatment, treatment, and direct inactivation) [77]. The antiviral mechanism may involve destruction of the enzyme system required for virus proliferation by the realgar nanoparticles, so that the virus cannot proliferate in living cells. Realgar nanoparticles may also directly kill the virus by directly destroying the virus envelope protein, thereby reducing virus viability [77]. The pharmacological mechanism of realgar is presented in Figure 2.

#### 3.1.2. Research on Realgar Formulas

*(1) Antitumor Mechanisms*. Since the 1970s, researchers in the field of blood diseases in China have successively applied realgar-*Indigo naturalis* formula (RIF) as the active ingredient in the clinical treatment of APL, achieving definite curative effects. RIF, a typical representative of realgar anticancer preparations, has now been approved for marketing in China.

A molecular-level study indicated that realgar is the principal component of the formula, whereas tanshinone IIA and indirubin serve as adjuvant ingredients [78]. Clinical trials have confirmed that oral arsenic (RIF) provides an outcome similar to that of intravenous arsenic trioxide (ATO) [79, 80]. More detailed clinical trials have shown that RIF is also safe and effective in newly diagnosed pediatric APL patients [81]. Furthermore, compared with ATO plus all-trans retinoic acid (ATRA), simultaneous use of oral RIF plus ATRA greatly reduces the clinical expenses [82, 83] and hospital stays [84–87] of APL patients during induction and remission therapy. According to long-termfollow-up results, RIF provides excellent long-term survival advantages [88]. The possible mechanisms of action include [78] (1) synergistic effects on human APL cell differentiation in vitro, (2) enhanced ubiquitination and degradation of the PML-RAR*α* oncoprotein, (3) relief of transcriptional suppression, and (4) G1/G0 arrest of APL cells with effects on key regulators of cell cycle progression. The main component realgar cooperates with other components in RIF to increase the absorption rate by inducing upregulation of the transmembrane protein aquaglyceroporin 9. Further studies have shown that RIF can significantly inhibit the growth of APL NB4 cells [89] and the proliferation and viability of the K562 cell line [90].

Clinical studies have shown that the Chinese medicine Qinghuang Powder (QHP) (daily dose of 0.1 g of realgar), the main component of which is realgar, is effective and safe for the treatment of patients with MDS and that reasonable adjustment of the daily dose of realgar can improve the efficacy without increasing the clinical toxicity [8, 9]. In vitro studies have shown that realgar, as the main drug contained in QHP, can induce cell differentiation in AML cells that progress from MDS, which might explain one of the mechanisms of QHP in the treatment of MDS [91].

Different DNA methylation subtypes may be present in MDS-metachromatic leukodystrophy (MLD) patients. A clinical study found that Chinese herbal medicine containing realgar has good effects in the treatment of MDS-MLD patients with the hypomethylation subtype [7]. The therapeutic mechanism may involve an increase in DNA methyltransferase expression in MDS [6]. After genotyping 43 MDS patients via ultradeep targeted sequencing, Zhao et al. [92] found that QHP was effective and safe, especially in those with genetic mutations in SF3B1, DNMT3A, U2AF1, and/or ASXL1. Another study revealed that As₂S₂ can reduce BCR-ABL protein levels in chronic myelogenous leukemia cells without affecting its transcription level. The mechanism of action involves binding of As₂S₂ to the RING finger domain of CBL (a RING-type E3 ligase) to inhibit its self-ubiquitination/degradation, thereby ubiquitinating the BCR-ABL protein [93].

These findings suggest that traditional Chinese medicine products containing realgar represent potential drugs with multiple activities. The active ingredients of realgar can be combined with some antitumor drugs to synergistically enhance the inhibition of tumor cells and reverse antitumor drug resistance [8], thus enhancing therapeutic efficacy [94].

*(2) Antiviral and Anti-Inflammatory Mechanisms*. Modern physicians have also applied realgar to various conditions. Professor Liangchun, a master of traditional Chinese medicine, used the traditional Chinese medicine formula Duo-Tan-Ding-Jing-San created with realgar to treat viral infectious diseases such as Japanese encephalitis, pneumonia, and meningitis and achieved good clinical results [95].

The realgar-containing Chinese medicine formula Liu Shen Wan (LSW) can significantly inhibit the influenza virus at different stages of viral replication in vitro. The antiviral effect is attributable to downregulation of the expression of inflammatory cytokines induced by the influenza virus via regulation of the activity of the TLR4/NF-*к*B signaling pathway [96]. Further research has shown that LSW significantly ameliorates lung injury caused by viral and secondary bacterial infections [97].

### 3.2. Toxicological Research on Realgar

Studies have shown that the disturbance of the intestinal flora and the severity of intestinal inflammation in realgar-treated mice are related to the realgar dose [98]. Therefore, in clinical applications, realgar should be used at a low dose in combination with other drugs to reduce intestinal inflammation [98].

One study performed untargeted lipidomics and other analyses on realgar-exposed mice to identify markers of the toxicity of realgar exposure to the nervous system [99]. The study found that the arsenic contained in realgar passed through the blood-brain barrier and accumulated in the brain, causing abnormal changes in the cerebral cortex [99]. In addition, increased oxidative damage and lipid dysfunction are also responsible for the neurotoxicity of realgar [99]. The specific mechanism involves excessive activation of Nrf2 regulated by the upstream signaling molecules ERK1/2 and p38MAPK [100].

Metabolomics studies have revealed that oral administration of realgar disrupts endogenous metabolites and related metabolic pathways in mice [101]. Follow-up kidney proteomic studies have revealed that apoptosis and oxidative stress might be related to realgar-induced nephrotoxicity in mice [102].

Cytochrome P450 enzymes are extremely important enzyme transport systems in the body. The main function of these enzymes is to metabolize exogenous substances (such as drugs) and endogenous substances. However, some studies have shown that higher doses of realgar can inhibit the enzymatic activity of cytochrome P450 enzymes, which may be related to the accumulation of arsenic from realgar [103]. Realgar exposure in mice activates innate immune-mediated inflammatory responses and disrupts the homeostasis of bile acids in the liver, plasma, and urine, causing immune-inflammatory infiltration in the liver [104]. A cross-sectional study of 1556 adults from the area surrounding a realgar plant showed that chronic arsenic exposure impairs cognitive function in adults and that this impairment is positively dose-related [105].

Although the toxicity of realgar nanoparticles is lower than that of realgar, studies have shown that realgar nanoparticles can induce free fatty acid and triglyceride accumulation, resulting in hepatotoxicity, and the metabolic markers of nanoparticle-treated subjects are different from those of traditional realgar-treated subjects [106].

Long-term or high-dose consumption of realgar can lead to the accumulation of heavy metals in the body, causing damage to the liver, blood, and nervous system. However, some clinical studies have found that higher concentrations of As₂S₂ in peripheral blood can enhance the clinical efficacy of realgar in the treatment of MDS-MLD patients [107]. Therefore, it is hypothesized that the pharmacological activity and toxicity of realgar may differ according to the activity of different cells. Realgar has a protective effect on nerve cells under pathological conditions but is harmful to normal nerve cells.

## 4. Research on Formulas Containing Both Cinnabar and Realgar

Compared with single mineral medicines, traditional Chinese medicine formulas containing multiple mineral medicines are more widely used in clinical practice. The mechanisms of action of mineral medicines or mineral-containing medicines in the treatment of diseases are complex but are related to the multicomponent, multitarget, and multichannel treatment characteristics of the medicines; these features also represent the characteristics and advantages of TCM for the treatment of diseases in general. Cinnabar and realgar are often used together in traditional Chinese medicine compounds.

### 4.1. Basic Medical Research

An-Gong-Niu-Huang Wan (AGNH) is a famous traditional Chinese medicine used to treat cerebral emergencies, such as traumatic brain injury, hemorrhage, brain infection, ischemia, and stroke, with a history of use of more than 3,000 years [1]. Modern research has suggested that AGNH has neuroprotective effects by reducing infarction volume, preserving blood-brain barrier integrity, and improving neurological functions against cerebral ischemia-reperfusion injury [108].

Based on skepticism about mineral medicines, some scholars have tried to remove cinnabar and realgar from AGNH. Interestingly, removing realgar and/or cinnabar from AGNH abolishes the neuroprotective effects [108]. In one study, histopathological analysis of mice that received AGNH orally showed that AGNH caused mild or no injuries to the liver and kidneys [109].

Wu Jutong, a warm-disease scientist of the Qing Dynasty, called Angong Niuhuang Pill the first of the “three classic antipyretic preparations” and believed that it had good heat-clearing, detoxifying, and sedating effects. Modern research suggests that cinnabar and realgar in the Angong Niuhuang Pill may play the main roles in “clearing heat and detoxifying” by helping inhibit the excessive release of inflammatory mediators [110]. This formula has significant effects against acute cerebrovascular disease, the Japanese epidemics of encephalitis and cerebrospinal meningitis, acute jaundice and primary hepatitis, renal failure, severe pneumonia, and infantile measles [110]. Tang et al.'s study on a fever rat model showed that realgar overinduced stress proteins (HSP70, HO-1) to improve positive stress levels in the body and inhibited overrelease of some inflammatory mediators (IL-1beta) to reduce inflammatory reactions under pathological conditions and inhibit the storm of inflammatory factors, thereby helping remove internal toxins and prevent damage to important organs [111].

Some scholars [112] have conducted similar research on Hua-Feng-Dan (HFD). Traditional HFD (containing 10% cinnabar and 10% realgar) improves behavioral dysfunction and attenuates microglial activation, rescuing the loss of dopamine (DA) neurons. However, a HFD lacking cinnabar and realgar is ineffective. This phenomenon reveals that cinnabar and realgar are active ingredients in HFD that exert useful effects in a neurodegenerative model and on the gut microbiota.

### 4.2. Clinical Applications

AGNH and HFD are still widely used clinically in their traditional and new forms. These agents can be used alone or in combination with other drugs for synergistic treatment.

Some meta-analyses of clinical studies on AGNH have revealed that AGNH has been widely used for acute cerebral infarction and intracerebral hemorrhage [113], traumatic brain damage [114], persistent vegetative state [115], and other severe cranial brain diseases. Moreover, studies have found that it can effectively promote postoperative recovery and exert respiratory care effects in chronic obstructive pulmonary disease patients after cardiac surgery [116]. However, there are relatively few clinical reports on the therapeutic effects of HFD, and all of the existing reports were published in China. HFD is often used in combination with other drugs in clinical applications and has good therapeutic effects on epilepsy [117], stroke [118], peripheral facial paralysis [119], and idiopathic facial paralysis [120].

With the introduction of modern biopharmaceutical technologies, innovations in the dosage forms of traditional Chinese medicine, such as capsules or liquid forms, have been reported. Based on these innovations, the pharmacological effects of the medicines are relatively clear, and these formulations have achieved outstanding curative effects that have been widely recognized in the medical field in recent years.

Traditional Chinese medicine injections are unique dosage forms in mainland China [121] that are administered by intramuscular injection or intravenous infusion. Qingkailing (QKL) injection is based upon the traditional Chinese medicine formulation AGNH, which is widely used in the treatment of many diseases given its good clinical efficacy. The main target diseases include acute cerebrovascular diseases and respiratory system infections, such as upper respiratory tract infection [122], pneumonia caused by respiratory syncytial virus [123], and acute stroke [124]. In the 2003 SARS incident, QKL was used as the basic drug for the treatment of atypical pneumonia with the integration of traditional Chinese and Western medicines and was combined with other drugs for comprehensive treatment [125]. During the coronavirus disease (COVID)-19 pandemic, Beijing also included QKL capsules in its clinical treatment plan [126].

In a previous study, QKL (or combined treatment) had a better effect on most outcome indicators compared with control, but the occurrence of adverse reactions cannot be ignored [127]. The current evidence, while being weak, indicates that QKL carries a low risk of adverse drug reactions and adverse events; however, some of the adverse events may be ascribed to improper use of the drug [128]. In the future, macromolecular substances should be removed as thoroughly as possible in production [121]. Serious care should be taken when QKL is administered to children, and QKL should not be combined with cephalosporin [121]. In addition, studies have shown that the delivery of Qingkailing by nebulization shows an equivalent or better curative effect with fewer side effects than injection of QKL in the treatment of pneumonia, respiratory tract infection, and tracheitis [129].

Xingnaojing injection (XNJ) is the only Chinese herbal injection approved for emergency treatment of stroke in China [130]. In the treatment of ischemic stroke, XNJ results in a significantly better overall response rate and better improvement of clinical symptoms than conventional treatment alone [130, 131]. In addition, XNJ can be used alone or as an adjuvant therapy to reduce brain damage and improve nerve health for the treatment of acute cerebral hemorrhage [132] and traumatic craniocerebral injury [133, 134]. During the COVID-19 epidemic, the National Health Commission of the People's Republic of China listed XNJ as a recommended traditional Chinese medicine injection in the “Guidelines for the Diagnosis and Management of COVID-19 (8th Edition)” [135]. For patients with severe symptoms, the Angong Niuhuang Pill is also recommended [135].

The above findings clearly show that the application of new technologies and new processes in development and production processes has enabled innovations and improvements of TCM preparations. This new technology has not only driven the development of traditional Chinese medicine, but also (and more importantly) promoted the internationalization of traditional Chinese medicine. In the Chinese Pharmacopeia (2020) [1], 24 recipes contain both cinnabar and realgar, such as Jufang Zhibao Dan for the treatment of febrile diseases and Qingyu Piwen Dan for heat stroke. Elucidation of the pharmacology, safety, and clinical application of AGNH and HFD and their novel formulations is critical to help assess the benefits and risks of these metal-containing traditional medicines.

## 5. Safe Application of Cinnabar and Realgar

Chinese materia medica containing cinnabar and realgar are generally relatively nontoxic at therapeutic doses under the pharmacopeia guidelines [1]. Important factors affecting the toxicity of cinnabar and realgar include the processing method, dose, compatibility, and attenuation.

### 5.1. Processing Method

In TCM, cinnabar and realgar are subjected to grinding and washing (called Shui-Fei) at least 3-4 times, which is crucial for the safe use of cinnabar and realgar in medicine.

Ancient physicians emphasized several important purposes of concocting mineral medicines using the grinding and washing method: to remove impurities, to reduce soluble elements as much as possible, to control the temperature to avoid the formation of new toxic and harmful substances, and to make the drug powder extremely fine and improve its bioavailability. Modern research illustrates that, as a TCM processing method, water grinding reduces toxicity [136, 137].

### 5.2. Appropriate Doses

The 1995 edition of the Chinese Pharmacopoeia reduced the daily dose of cinnabar from 0.3 to 1.5 g to 0.1∼0.5 g, and this standard is still used today [1]. In the 2000–2020 editions of the Pharmacopoeia of the People's Republic of China [1], the dose of realgar was 0.05∼0.1 g. Studies have shown that the toxicity of cinnabar [19] and realgar [138] is concentration- and time-dependent. Therefore, mineral medicines should not be used in large quantities, nor should they be used in small amounts for a long time. It is also necessary to pay attention to the administration period and special populations. Use should be limited to special populations, such as children, pregnant women, and those with liver and kidney insufficiency.

### 5.3. Toxicity-Attenuating Compatibility

In TCM, minerals are not applied alone but mixed with herbs and/or animal products. Chemical reactions may occur due to the mixing of agents, and it is assumed that the various components promote each other to achieve the desired therapeutic effect and reduce toxicity [29]. Studies have confirmed that the toxicity of cinnabar is low and that the herbal medicines in cinnabar formulas can attenuate the damage caused by cinnabar to body systems [29, 139, 140], which may be related to the absorption, distribution, and excretion of Mercury [28, 138]. Similarly, when realgar is used in a formula, the other herbal medicines in the formula can significantly reduce realgar toxicity by reducing blood arsenic levels [29, 141].

Metabolomics studies have confirmed that the other herbs present in AGNH alleviate inflammatory cell infiltration and damage in the liver and kidneys as well as the serum metabolic profile disruptions induced by cinnabar and realgar insults [139, 142]. The mechanism may involve protective effects of AGNH's herbal constituents against the accumulation of Hg and As and the hepatorenal toxicity induced by cinnabar and realgar via downregulation of the expression of uptake transporters and upregulation of the expression of efflux transporters in hepatorenal tissues [141]. Similar studies on LSW [143] and Niuhuang Jiedu tablets [144] have also demonstrated that other herbal ingredients in the formulas can reduce the damage caused by realgar to some extent.

## 6. Summary and Outlook

The above review demonstrates that, despite great progress in research on the pharmacological and toxicological mechanisms of cinnabar and realgar, limited pharmacological studies have assessed cinnabar's sedative and soothing effects and realgar's anti-inflammatory, antioxidant, and epidemic-preventing effects. Notably, the compositions of metal mineral medicines are complex, as these medicines contain a variety of compounds. However, research on the physiological effects of the other components is relatively limited. In addition, because the body itself contains various trace elements, it is difficult for the current analytical technologies to achieve accurate detection in vivo, which is the key hindrance to in-depth research on the absorption and metabolism of metal-based mineral drugs. Researching pharmacology, toxicology, and mechanisms of action is an important method to study medicinal properties. However, most of the research conducted to date has focused on the overall efficacy and toxicity of cinnabar, realgar, or their formulations. Many researchers are observing and testing some macro-indicators, but research to clarify the mechanisms of action at the molecular-level remains lacking. There is no sufficient scientific basis for rational clinical drug use, and there is an urgent need to conduct more in-depth research on the molecular mechanisms of the pharmacological and toxic effects of cinnabar and realgar.

To date, research on the toxicity of mineral drugs has mostly adopted modern toxicology methods to analyze the toxicity to normal organisms. However, due to neglect of the toxicity-reducing compatibility theory of traditional Chinese medicine, the toxicity of mineral medicines has been exaggerated.

To improve efficacy and reduce adverse effects, TCM often combines mineral medicines with herbs, animal products, and/or other minerals in products called formulas. Increasing evidence demonstrates that treatment regimens for various illnesses contain multiple drugs with distinct but related mechanisms. These drugs can often amplify the therapeutic efficacy of the other drugs, enabling multitargeted synergistic therapy and leading to maximal therapeutic efficacy with minimal adverse effects. However, the essential compounds have not been identified in most formulas, and the precise mechanisms of the formulas remain to be addressed using molecular approaches. When the specific mechanisms of action and toxicity mechanisms of two-drug formulas are unclear, the use of each single drug may not completely explain the pharmacodynamic and toxicity mechanisms of the two drugs together. Each drug may need to be combined with other compatible drugs to completely explain the possible mechanisms of action, and pharmacodynamic research may need to be conducted to achieve attenuation and antiviral effects.

For clinical applications, it is necessary to combine theory and clinical experience to guide scientific and rational drug use and achieve the purposes of reducing toxicity and increasing efficacy through the use of modern preparation methods or drug combinations. Processing has a significant effect on the content of toxic impurities in mineral medicines. The processing parameters of the washing method should be further standardized, and the amount of water added and the numbers of repeated grinding operations should be clarified to obtain decoctions of uniform quality. The development of nanopreparations, microbial leaching preparations, new crystal preparations, and compound preparations represents the current focus of research on mineral pharmaceutical preparations. How to effectively improve drug solubility, bioavailability, and targeting, adjust sustained release and controlled release, and reduce the occurrence of adverse reactions is the key issue to be considered with these new processing methods.

The next steps are to explore the effective traditional formulas through the combination of minerals and other drugs, to elucidate the mechanisms of these formulas, to upgrade the formulas into modern dosage forms, and to describe the traditional theories of traditional Chinese medicine, such as synergistic treatments and toxin counteraction with toxin therapy, in the modern language of molecular biology. The complementary combination of systems biology and theoretical research on TCM will greatly promote the pace of traditional Chinese medicine internationalization.

## Supplementary Materials

Supplementary Materials The authors review the accumulating evidence and discuss the pharmacology, toxicology, formulas, and safe use of cinnabar (HgS) and realgar (As₂S₂).
