# Supplementary figures and images for: Pharmacology, Toxicology, and Rational Application of Cinnabar, Realgar, and Their Formulations
Source: Evid Based Complement Alternat Med. 2022 Sep 27;2022:6369150. doi: 10.1155/2022/6369150 (PMC9532072; doi:10.1155/2022/6369150)

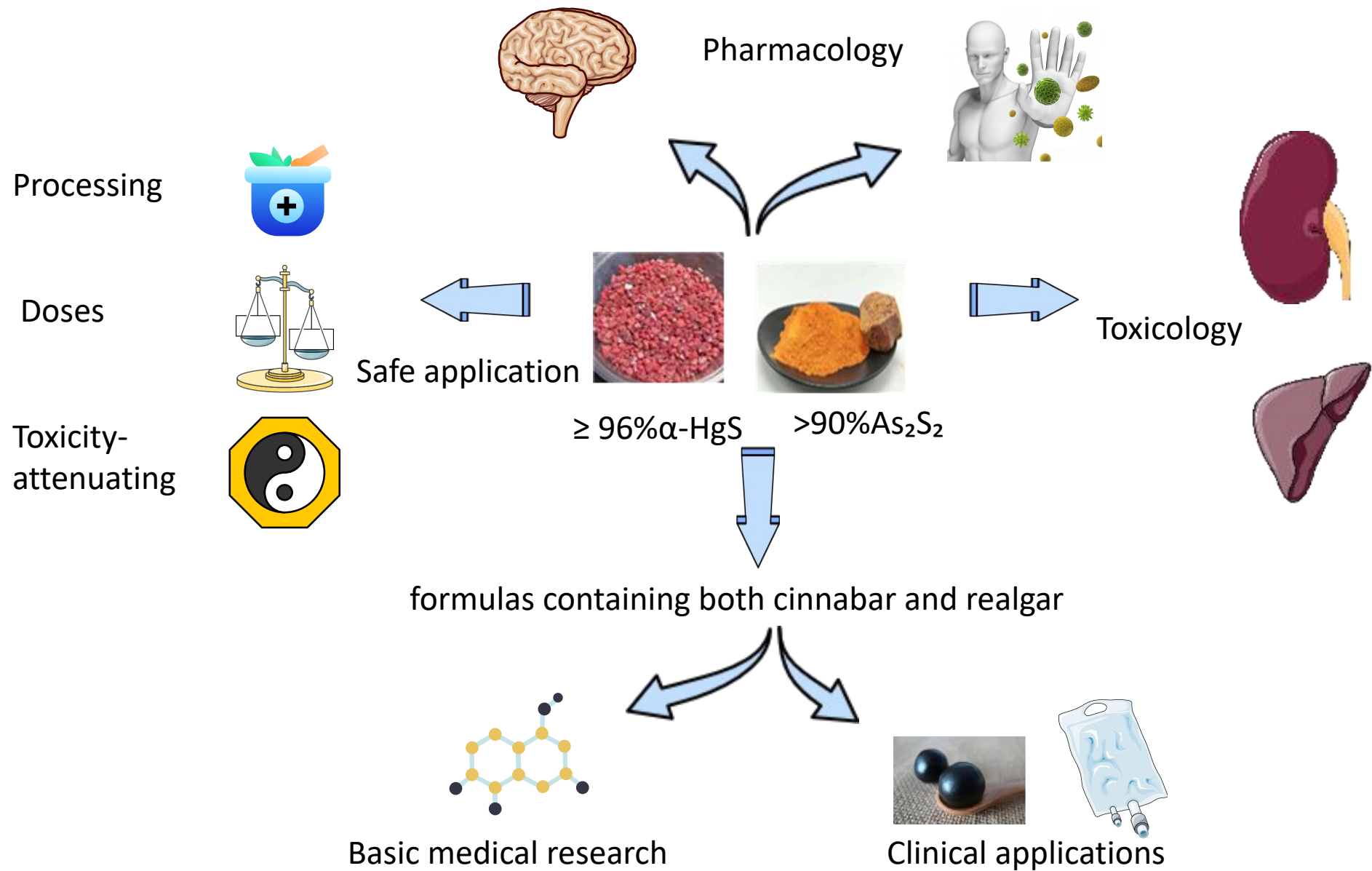

Supplement: Supplementary Materials — The authors review the accumulating evidence and discuss the pharmacology, toxicology, formulas, and safe use of cinnabar (HgS) and realgar (As₂S₂). [file 6369150.f1.pdf]
